# YOU’VE GOT A FRIEND: SOCIAL FACTORS AND DEPRESSIVE SYMPTOMS IN OLDER ADULTS

**DOI:** 10.1093/geroni/igac059.3002

**Published:** 2022-12-20

**Authors:** Erika Fenstermacher, Amy Fiske

**Affiliations:** West Virginia University, Pursglove, West Virginia, United States; West Virginia University, Morgantown, West Virginia, United States

## Abstract

Depression in late life is associated with disability, lower quality of life, increased mortality, and risk of suicide. Research suggests that functional disability, which is often brought about by a medical condition, may precede depressive symptoms and may be a major factor for older adults developing depression. Social support can be beneficial for both emotional and physical health. Numerous studies demonstrate that social networks, perceived and subjective social support, and satisfaction with support received moderate the relation between health problems and depression. Findings are mixed regarding received and objective social support and only one study was found that examined the impact of giving support to others. The current study addressed gaps in the literature by focusing on three facets of social relationships: availability of support, participant giving help, and participant receiving help. Data from the 2011 wave of the Wisconsin Longitudinal Study were used (N = 3906; mean age of 71.27; SD = .948). We examined the relationships between medical conditions, functional impairment, depressive symptoms, and social support using regression analyses. Medical conditions and functional impairment each independently predicted depressive symptoms. Availability of support significantly predicted lower levels of depressive symptoms and amount of help received predicted higher levels of depressive symptoms, controlling for medical conditions and functional impairment. These findings suggest that simply having someone available, not necessarily interacting with them for help, could reduce depressive symptoms in older adults.

